# In-House 3D Printed Positioning and Cutting Guide System for Mandibular Reconstruction. Protocol and case report

**DOI:** 10.4317/jced.61278

**Published:** 2024-02-01

**Authors:** Juan-Pablo Rodríguez-Arias, Álvaro-Damian Moreiras-Sánchez, Alessandro Gutiérrez-Venturini, Marta-María Pampín, Javier González, Maria-José Morán, José-Luis Del Castillo, Carlos Navarro-Cuéllar, José-Luis Cebrián

**Affiliations:** 1 Physician attending / Faculty. Oral and Maxillofacial Surgery Department. La Paz University Hospital. Madrid, Spain; 2 3D Management Laboratory, La Paz University Hospital, 28046 Madrid, Spain; 3 Physician attending / Faculty. Oral and Maxillofacial Surgery Department. Gregorio Marañón University Hospital. Madrid, Spain; 4 Head of the Department. Oral and Maxillofacial Surgery Department. La Paz University Hospital. Madrid, Spain

## Abstract

Maxillofacial surgery planning has been improved by technological advances in 3D printing. The use of customized cutting and positioning guides allows intraoperative reproduction of pre-planned osteotomy cuts, resulting in increased surgical accuracy, reduced surgical time and improved esthetic and functional outcomes. Our paper presents a new method for creating and printing in-house cutting and positioning guides. A computer program (Brainlab iPlan) was used to segment the mandible for three-dimensional planning from imported conventional computed tomography (CT) scans. The virtual model of the mandible was printed on a stereolithography (SLA) 3D printer and a reconstruction plate was adapted to the printed model. The surface of the model and the screw-retained plate was scanned using a structured light surface 3D scanner (Artec Eva). The obtained scan of the jaw and plate in position was processed and transformed into an STL file. Free software (Autodesk Meshmixer) superimposes the initial jaw on the scanned jaw with the plate, designing a customized hybrid cutting guide that allows accurate intraoperative positioning, knowing the exact position of the reconstruction plate screws in the jaw. The total design, fabrication and 3D printing time for the in-house hybrid guide was 595 min. The average total printing cost was EUR 16. We found the technique to be simple and repeatable. We present and describe here a novel and simple technique for in-house 3D printed positioning and cutting guide system which can be applied to overall maxillofacial area. In cases of mandibular reconstruction, this protocol guarantees an adequate esthetic and functional result.

** Key words:**Oral cancer, 3D surgery, CAD/CAM, personalized medicine, surgical guides, in house.

## Introduction

As is known, the craniomaxillofacial region (CMF) is characterized by its complex three-dimensional (3D) anatomy. This is why it is sometimes difficult to ensure the patient’s quality of life, both in terms of aesthetics and function.

In the quest to improve the quality of life of our patients, the application of Computer Assisted Design - Computer Assisted Manufacturing (CAD/CAM) technology in maxillofacial surgery has become routine. Whether in oncologic and reconstructive surgery, orthognathic surgery, or traumatology, the use of cutting guides or pre-curved plates, or patient-specific cutting guides and osteosynthesis plates, has become widespread and standardized due to their widely demonstrated safety and cost-efficiency (1-3).

However, as a consequence of the economic crises suffered, public health systems are under pressure to reduce costs, so doctors and surgeons must ensure that patients receive the best possible treatment with the means available while acting as good stewards of those means. The fact that patient-specific implants (PSI) , such as cutting guides or plates, are mostly offered and produced by osteosynthesis manufacturing companies raises the question of whether their use has really led to an overall reduction in costs, despite the fact that several articles report it as a self-financing technology (1-3). Consequently, to avoid this dependency, in-house virtual planning has become an important part of our maxillofacial Point-Of-Care (POC) manufacturing workflow (4). The advantages of an in-house system have been reported, such as faster prototyping and planning, higher cost-effectiveness and faster manufacturing.

In cases of mandibular reconstruction, correct restoration of the mandibular contour is crucial when reconstruction is done using a plate, either with a free or vascularized graft or simply with the plate alone. For this, the three-dimensional relationship between the intermaxillary occlusion and the placement of the condyles within the glenoid fossa must be considered. Systems and protocols have been described to try to improve accuracy, such as the design of a 3D printed space maintainer for plate positioning (5,6) or the creation of rudimentary positioning guides, making the holes in the cutting guide before sterilization (the cutting guide is applied onto the biomodel, and holes created manually in the cutting guide to correspond with those on the pre-bent plate) (7,8). Moreover, there are several in-house designs of cutting guides for mandibular resection osteotomies, however, usually the POC of each hospital does not have the option of 3D printing titanium and it is in this situation where we can find the biggest accuracy errors. These would be solved by including the final plate holes in the cutting guide, also becoming a positioning guide.

In this article, we propose a new protocol, which requires a scanning system to acquire the model of the mandible with the preformed plate screwed in its planned position and, afterwards, create and 3D print the cutting and positioning guide, which presents the holes of the reconstruction plate in its exact position. We believe that this protocol could be applied to any surgery requiring customized guides and plates.

## Case Report

**Figure 1:**
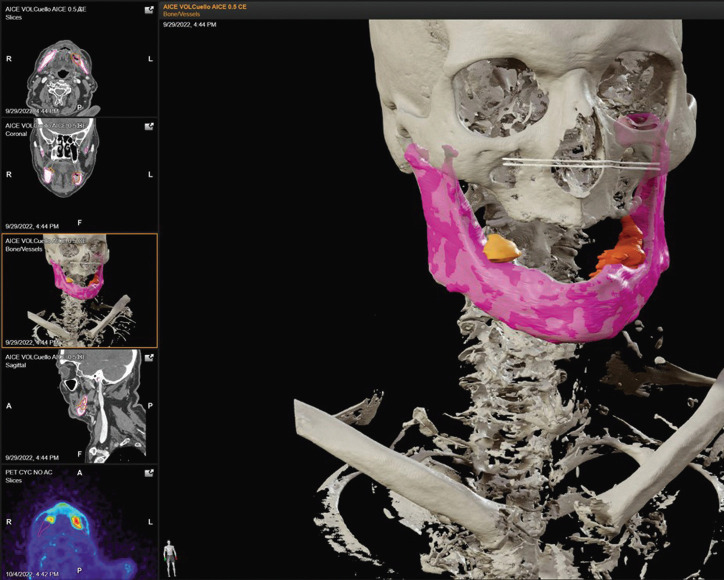
Superimposition of PET and CT in Brainlab. Segmentation of the mandible and the two le-sions to treat. In this case, the lesion located in the left mandibular body (orange) will be guided.

In this section, the proposed protocol is explained, and divided into different stages:

We present the case of an 84-year-old male who presented two simultaneous squamous cell carcinomas in both mandibular bodies, the one located in the left mandibular region being larger than the other, (Fig. 1). In this case, the guidance was performed for the latter, where a segmental mandibulectomy was planned.

-Virtual Planning

Thin-slice maxillofacial CT scans of the patients were downloaded as Digital Imaging and Communications in Medicine (DICOM) data, from the hospital’s imaging software. The imaging protocol used 0.625 mm slice thickness for the bone windows to allow for the highest definition of the images.

DICOM files of the preoperative CT scan of the patient were imported to the Brainlab iPlan® software, which allows seg-mentation of regions of interest and obtaining STL (Standard Tessellation Language) files.

In our case, CT and PET images were superimposed in Brainlab iPlan. The mandible was segmented, obtaining its STL file, as well as the lesions to be treated and the necessary margin, according to the tumor nature (Fig. 1).

STL files were imported into 3D Slicer software that also allows segmentation of regions of interest as well as processing and printing.

-3D Model Printing and Plate Shaping

The virtual model of the mandible was printed in a stereolithography (SLA) 3D printer (Form 2, Formlabs, Somerville, MA, USA) with Tough 1500 Resin (Formlabs) at a 0.1 mm printing resolution.

**Figure 2:**
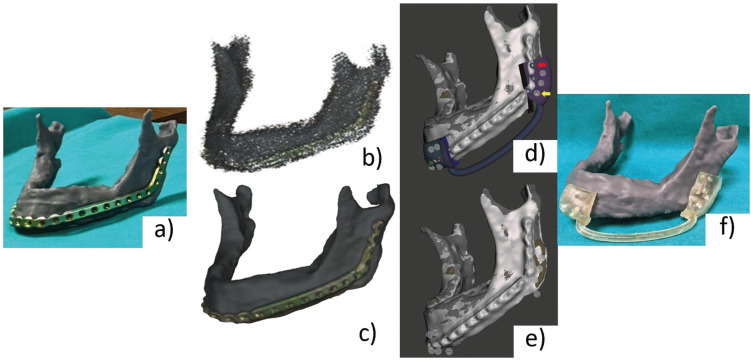
(a) 3D model of the jaw, with the adapted plate screwed. (b) Surface scanning of the model and screwed plate point cloud data before post-processing; (c) Final reconstruction of the 3D digital model after post-processing. (d) Su-perimposition of the STL of the initial segmentation of the mandible with the STL of the scanned jaw and plate. (e) Superimposed mandibular STLs and hybrid guide. Noted the holes that will temporarily fix the mandible to perform the osteotomy (yellow arrow) and those that will fix the plate, which are raised (red arrow). (f) 3D printed mandible and hybrid guide.

A 3.0 profile reconstruction plate (KLS Martin, Tuttlingen, Germany) was adapted to the printed model preoperatively, and subsequently sterilized for intraoperative use. The printed mandibular model served as a nonsterile intraoperative reference (Fig. 2a).

In our case, the lesion did not convex the mandibular cortex. In case of protrusion, the 3D printed model should not contain the complete model, but rather the model of the mandible with the planned osteotomy and a space maintainer or the planned fibula reconstruction, so that the plate can be molded over it.

-Surface scanning of the model in conjunction with the bolted plate

High accuracy is needed to capture the detailed positioning of holes, which is essential for a successful reconstruction.

Hence, a structured-light surface 3D scanner (Artec Eva, Artec Group, Luxembourg) was used. This scanner can record the model’s texture and capture surfaces with a 3D resolution of up to 0.2 mm.

The obtained point cloud data were processed in the native Artec Studio 16 Professional software (Artec Group, Luxembourg), following different steps. The point cloud data’s undesired regions, such as the background, can be manually removed using the erasing tool. The registration tool then examines and aligns each partial scan that was captured during the scanning procedure. The excess points surrounding the object are eliminated when the outlier removal tool is applied. A single mesh 3D model is obtained from the fusion of all the partial scans of the point cloud data, and texture from the initial scan can be applied to it

Figure 2b shows the original scan and the final textured mesh, exported as an STL file for the following step.

-Creation of the hybrid guide

The exported STL of the mandible and plate in position was then processed.

First, the mandible was refined, removing the noise and filling the defective areas, in order to obtain a closed and clean mesh. Next, this mesh was superimposed on the previously segmented STL of the mandible, using this latter as a reference. For this, we used the iterative closest point (ICP) tool in the free software Cloud Compare (CloudCompare v 2.12.3 Kyiv, Ukraine). This way we have the information of where the lesion is, for the creation of the cutting guides, as well as where the holes and screws of the plate should be, to add these holes in the cutting guide, making it both a positioning and cutting guide (Fig. 2d).

Afterward, the osteotomies were designed according to the extent of the lesion, with an adequate margin using the modeling software Meshmixer (v 3.5, Autodesk Inc., CA, USA). Subsequently, we used the “Offset” tool to design a guide that was placed on the vestibular and inferior surface of the proximal and distal mandibular segments. These were connected by a tube. Then, thanks to the scanned 3D model, in addition to creating the holes in the guide that will fix the guide to the mandible temporarily, we can also plan the definitive ones, which will fix the plate to the mandible, once the resection is done. This creates a hybrid guide, containing information both of the positioning of the plate and the osteotomies planned for tumor resection (Fig. 2e).

Lastly, this guide was exported in STL format for the 3D printing phase.

-3D printing of the guide

The digital guide was printed using Surgical Guide resin from Formlabs at a printing resolution of 0.1 mm using a stereo-lithography (SLA) 3D printer (Form 2, Formlabs, Somerville, MA, USA) (Fig. 2f).

After printing, the guide was taken off the build platform and cleaned of any remaining liquid resin by immersing them for 20 minutes in a Form Wash (Formlabs) filled with 99% isopropyl alcohol (IPA). It was then post-cured in a Form Cure (Formlabs) at 60°C for 30 minutes to obtain biocompatibility and the best mechanical properties. The guide was then sterilized and ready to use in the operating room. The guide was then sterilized and ready to use in the operating room.

**Figure 3:**
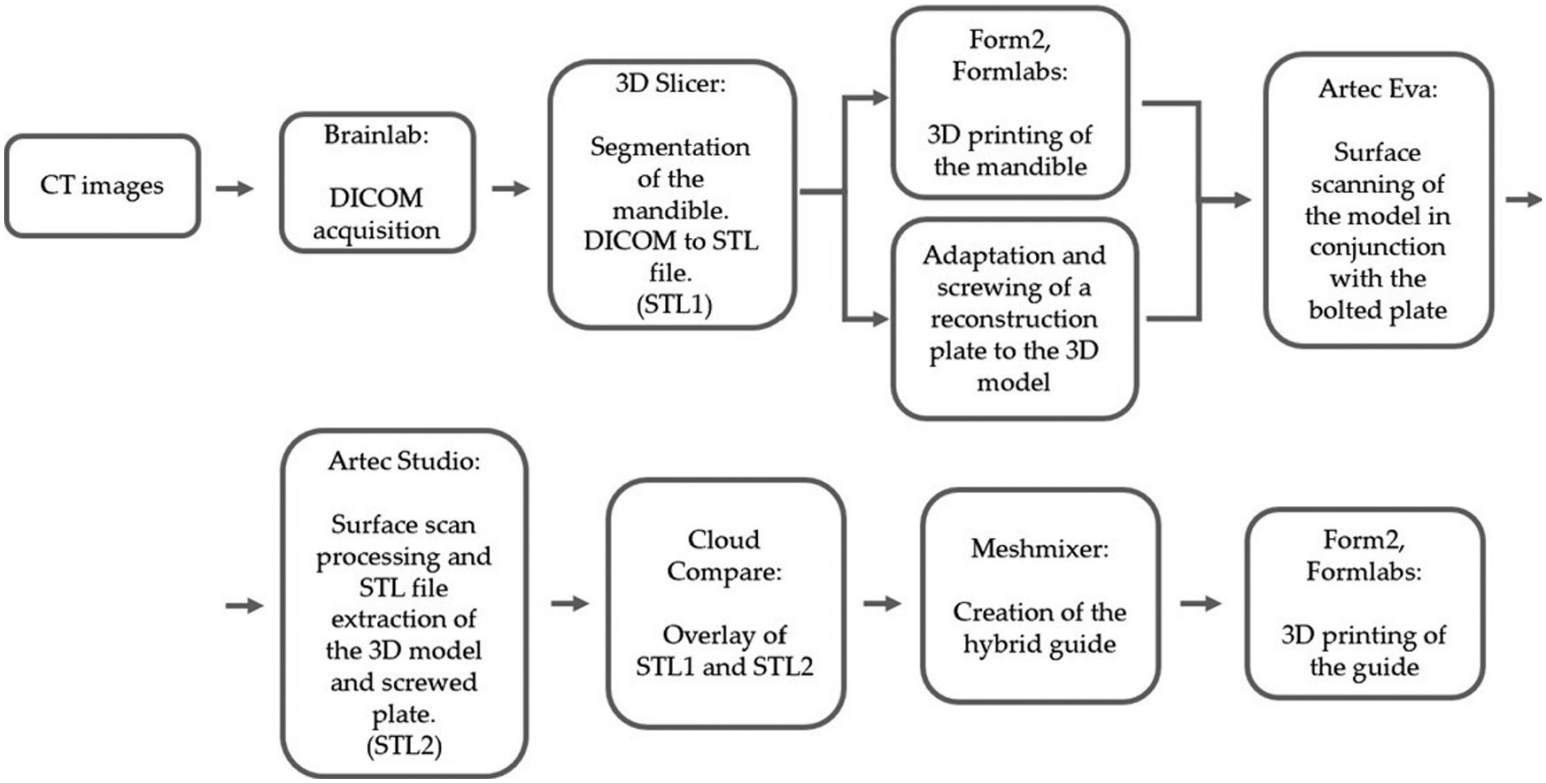
This workflow illustrates the process from imaging through planning to scanning, printing, and surgery.

The workflow has been simplified to make following the protocol easier (Fig. 3).

-Surgery

**Figure 4:**
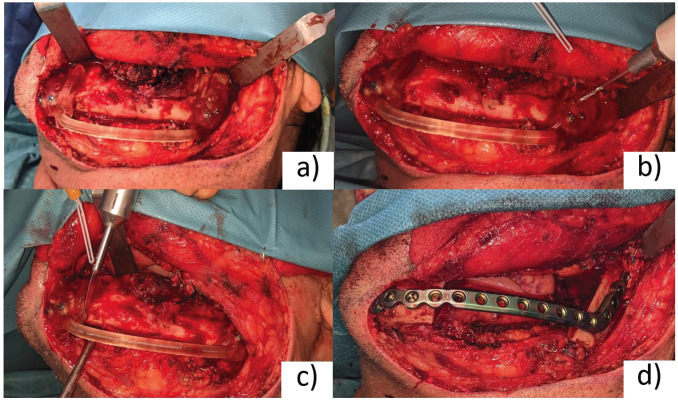
Intraoperative photographs of the left mandibulectomy performed. (a) Hybrid guide bolting, with a proper fit. (b) Screw holes were drilled to locate the position of the plate. (c) Performing the osteotomies, according to the guide. (d) The reconstruction plate was posi-tioned to match the previously drilled holes.

In the operating room, a left cervical approach was performed under general anesthesia. Subplatysmal undermining was done until the mandibular basal bone was reached. The facial vessels were identified and ligated. Once the left mandibular body was completely exposed, tumor resection was performed via a combined intraoral and cervical approach. The hybrid guide was placed and fixed with 2.0 screws (KLS Martin, Tuttlingen, Germany) (Fig. 4a). Screw holes marking the position of the plate were drilled in this step (Fig. 4b). Then, the osteotomies were performed and tumor resection was completed (Fig. 4c). The guide was removed and the pre-bent 3.0 reconstruction plate (KLS Martin, Tuttlingen, Germany) was positioned according to the previously drilled holes, fitting uneventfully over the remaining mandible (Fig. 4d). Adequate hemostasis was achieved and the wound was closed in layers and a suction drain placed.

The average time to extract the bone region of interest from the patient’s CT scan was 10 min, the time to print the first model was 300 min, and the time to prebent the plate was 15 min. The scanning of the model and plate was 10 min and its subsequent processing was 20 min. Finally, the time for virtual surgical planning and virtual design of the hybrid cutting and positioning guide was 120 min and its printing took 120 min. Interestingly, of the almost 10 hours needed, 7 hours were printing time.

The total cost per case of printing the mandibular model was EUR 6, and that of the cutting guides is EUR 10. This was calculated based on the amount of material and type of material used for printing calculated by the printing software, without taking into account the cost of the printer or scanner.

## Discussion

Restoring the mandible’s preoperative form and function is the goal of reconstruction after segmental excision. PSI, such as cutting guides or plates, for mandibular reconstruction, have been shown to improve bone reconstruction accuracy, overall surgical efficiency, and reconstruction precision (7-9).

Even though the advantages of using PSI are well known and that the third-party company fee can be offset by the reduction in the cost of managing complications and the increase in intraoperative time (1-3), the cost estimates for this technology, which range from EUR 3000 to EUR 5000 per patient, and a product delivery time of more than 3 weeks (7), prevent its widespread adoption. Moreover, reliance on osteosynthesis manufacturing companies to obtain the PSI requires the existence of a national operational infrastructure on their part, which, on the one hand, entails a cost overrun for the healthcare system and, on the other, generates dependence on the industry by reducing the flexibility of the planning schedule. Meanwhile, economic crises impose pressure on public health systems to cut costs, forcing physicians and surgeons to provide the best care feasible with the available resources while simultaneously acting as good stewards of these resources to safeguard patients’ well-being. Cost-cutting measures can be crucial for improving patient care.

To avoid this dependency and reduce costs, in-house virtual planning has become an important part of our workflow and it is becoming increasingly common for hospitals to set up their own 3D laboratories (FabLabs) to meet their 3D design and manufacturing needs, known as POC (4). Dell’Aversana Orabona in 2018 (10) and Sharkh in 2019 (11), found that the time needed to produce the personalized cutting guides using an internal process may be cut by up to 24 hours when compared to the time needed by the commercial system. In addition, the price of printing material per case was estimated at 3 euros and $18.01 respectively. The key expense was the overall price of the 3D printer (approximately $5,000 CAD for the SLA Form 2 printer).

Maintaining sufficient condylar position, anteroposterior dimension, and space between bone segments might be challenging after mandibular resection. This occurs especially in two circumstances. In edentulous patients, where not only the condylar reference is lost, but also the occlusion is not available as a guide, and in cases where a plate cannot be shaped in the vestibular contour due to the expansion of the vestibular margin. Additionally, the placement of the hand-pre-bent titanium plates may not be perfect since the anatomical landmarks on the surface of the bone near lesions are frequently imprecise. We consider this to be the main problem with in-house cutting guides, which are not true PSI.

Several in-house cutting guide designs are available for mandibular resection osteotomies. However, we have not found any in the literature that also contains the screw position information of the final reconstruction plate, which would make it a positioning guide and a true in-house PSI. When CAD/CAM planning is performed in-house, usually, a CT scan is performed, the mandible is segmented and customized cutting guides are made for the planned resection. Subsequently, the STL model of the mandible is printed and a stock plate is pre-molded on this template. This happens because the POC of each hospital does not usually have the option of printing titanium, which requires specific and expensive machinery, and it is in this case that we can find the biggest errors in segment positioning. Systems and protocols have been described to try to improve this, such as the positioning of the guides on the model, having the holes of the plate marked, and making the holes in the cutting guide before sterilization (5,6) or the design of spacers (7,8). This would be solved with this hybrid guide, by including the holes of the end plate on the cutting guide, making it also a positioning guide.

We describe an in-house PSI technique for maxillofacial reconstructive procedures, proposing a new protocol, for which a scanning system of the mandible with the preformed plate is needed, to subsequently create the cutting and positioning guide, which presents the holes of the reconstruction plate in its exact position.

Multiple scanning options are available with an accuracy of < 0.1mm (12). The structured-light surface scanners, as the one we use in our case, provide good accuracy, but it requires the model to be printed with an opaque non-glossy material, making it adequate for the scanning process. Another suiTable method for acquiring the model is to use photogrammetry techniques. This technology offers excellent accuracy and precision, which, depending on the device, provide indicated point accuracy ranging between 0.05 and 1.0 mm and a resolution between 27.9 and 68.3 polygons/mm2 (12,13). The proper scanning of the plate in the model, as a successful positioning of the holes in the cutting guide, depends on this. A study contrasting various systems discovered that more affordable and accessible systems (such as the iPhone) demonstrated remarkable outcomes, taking it as a substitute for more expensive systems (12). Subsequent publications that verified the systems’ accuracy and dependability with comparable acquisition times made them more available and practical for everyday application (14).

The procedure presented here we believe to be a novel and unique one, accessible to the vast majority of hospitals due to the use of 3D printers, which allow for a reduction of costs and manufacturing time. Furthermore, we do not consider the need for a surface scanner to be a limitation, due to the possibility of scanning with a cell phone in a very accurate way. In the case described, one of the most advantageous features of the device is its ability to correctly perform the desired mandibular resection during surgery and its ability to restore the preoperative position of the proximal and distal regions of the mandible, even when no teeth are present or by reconstructing it with free vascularized osteocutaneous fibula flap. We also think that this is a versatile protocol that can be used both for reconstructive surgery, as in this case, and for orthognathic or trauma surgery in the CMF region.

In our opinion, it would be quite interesting to compare our in-house hybrid cutting and positioning guides with those produced by osteosynthesis manufacturers overlapping preoperative and postoperative CT scans. Currently, we are working on this project, which we will publish as soon as possible.

We present and describe here a novel and simple technique for an in-house 3D printed positioning and cutting guide system for mandibular reconstruction, which can be applied to orthognathic surgery or traumatology of the overall maxillofacial area. In cases of mandibular reconstruction, this protocol guarantees an adequate esthetic and functional result.
